# PITX1 plays essential functions in cancer

**DOI:** 10.3389/fonc.2023.1253238

**Published:** 2023-09-29

**Authors:** Jingpu Zhao, Yongfeng Xu

**Affiliations:** ^1^ Affiliated Hospital of Weifang Medical University, School of Clinical Medicine, Weifang Medical University, Weifang, Shandong, China; ^2^ Abdominal Oncology Ward, Cancer Center, West China Hospital of Sichuan University, Chengdu, Sichuan, China

**Keywords:** PITX1, stem cell, noncoding RNA, cancer, apoptosis

## Abstract

PITX1, also known as the pituitary homeobox 1 gene, has emerged as a key regulator in animal growth and development, attracting significant research attention. Recent investigations have revealed the implication of dysregulated PITX1 expression in tumorigenesis, highlighting its involvement in cancer development. Notably, PITX1 interacts with p53 and exerts control over crucial cellular processes including cell cycle progression, apoptosis, and chemotherapy resistance. Its influence extends to various tumors, such as esophageal, colorectal, gastric, and liver cancer, contributing to tumor progression and metastasis. Despite its significance, a comprehensive review examining PITX1’s role in oncology remains lacking. This review aims to address this gap by providing a comprehensive overview of PITX1 in different cancer types, with a particular focus on its clinicopathological significance.

## 1 Introduction

Paired-like homeodomain transcription factor 1 (PITX1), also known as pituitary homeobox 1 (Ptx1), belongs to the highly conserved homeobox genes that encode sequence-specific transcription factors (SSTFs) widely reported to be involved in bone development and mitosis (1). Its characteristic feature is the conserved 180-bp DNA sequence encoding a DNA binding homeodomain of 60 amino acids (2, 3). Homeobox genes play a critical role in establishing cell identity during the spatial and temporal dimensions of animal growth and development (4, 5). Mutations in these genes can lead to substantial developmental abnormalities, including homeotic transformations that alter the identity of specific body structures (6). Given their early expression during embryonic development, the PITX gene family has been extensively studied in the context of organ and body development (7). PITX1 also regulates the pattern of hindlimb tissues and pituitary development (8, 9), and is the earliest expressed gene in the PITX gene family during E6.5 primitive streak formation in the mesoderm of the posterior embryo. It is located on human chromosome 5, spanning 6,495 bp with one transcript of approximately 945 bp and three exons. It is an early developmental determinant of the hindlimb and regulates animal development (10). For example, PITX1-deficient mice exhibit mandibular dysplasia, cleft palate, forked tongue, and a smaller anterior pituitary lobe with decreased cell numbers in the follicle-stimulating hormone (FSH) and thyroid-stimulating hormone (TSH) lineages, leading to lower FSH and TSH levels (11). In addition, the loss of PITX1 leads to significantly decreased expression of Foxp4, Snai1, Sox6, Sox8, and Sox9, which regulate cartilage development, as well as Cxcl12, Eya1, Mef2c, Nfatc2, and Six1, which have unusual significance in regulating bone development and muscle shaping. The loss of PITX1 results in mice having a significantly smaller pelvis, missing ilium, smaller long bones, femurs, tibias, and fibulas (7).

**Figure 1:**
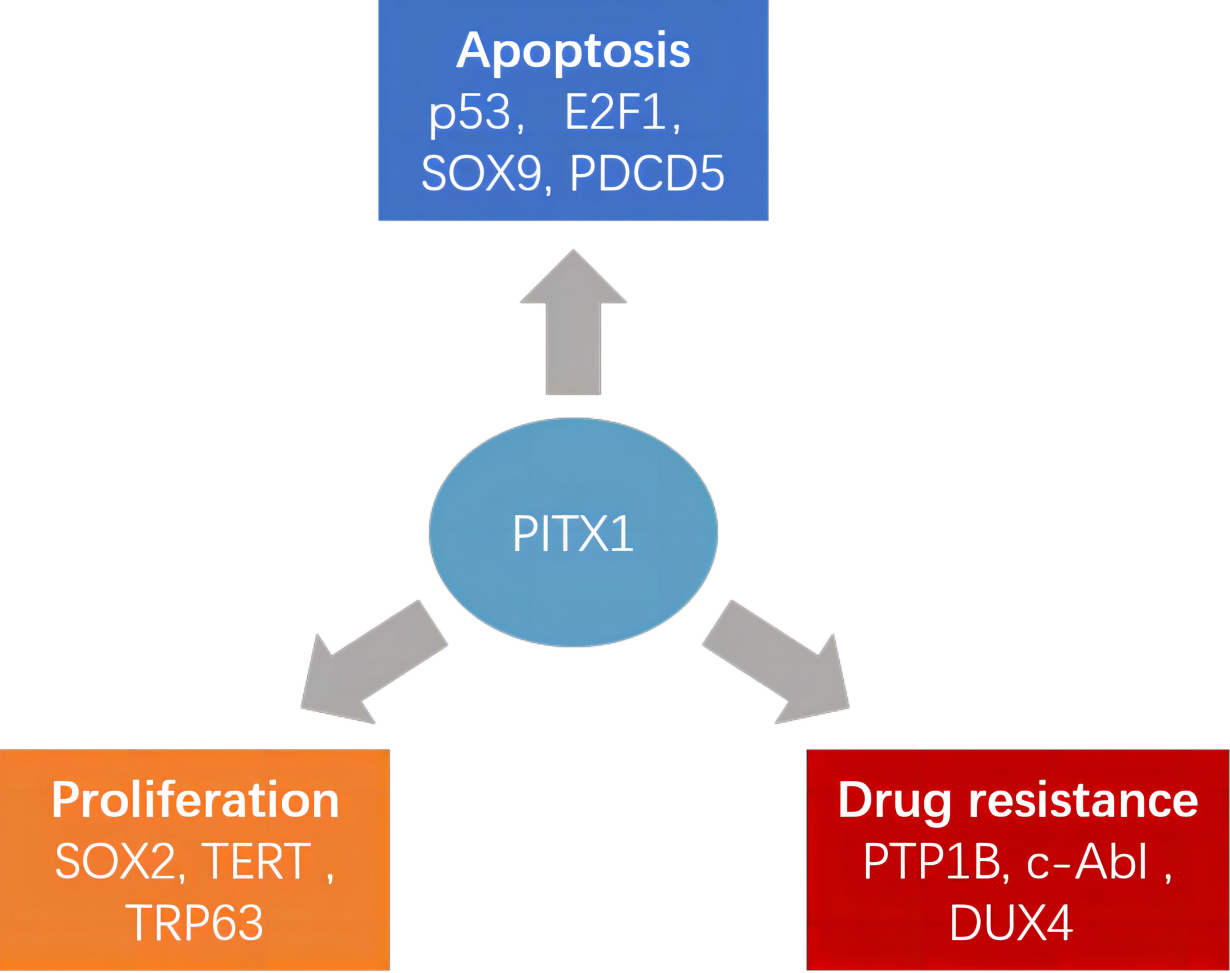
Different roles of PITX1 and its corresponding target proteins in tumors. PITX1 has been reported to play different roles in tumor proliferation, chemotherapy resistance, and apoptosis by activating various downstream effector factors.

Recently, the PITX1 gene has emerged as a notable player in the progression of diverse tumors, positioning it as a promising diagnostic marker and potential therapeutic target (**Figure 1**). Although many studies have reported the involvement of PITX1 in tumor formation and progression, there is still a lack of relevant reviews to summarize these findings. This comprehensive review delves into the multifaceted role of PITX1 in tumor formation. Subsequently, we extensively explore its involvement in tumor resistance, post-transcriptional modifications, noncoding RNA, tumor stem cells, and associated signaling pathways, shedding light on the intricate mechanisms underlying its contributions to cancer progression.

## 2 Current status of PITX1 in cancer research

Genetic alterations at the molecular or chromosomal level have been identified in most cancers, resulting in an imbalance between oncogenes and tumor suppressor genes that drives tumor growth, thus characterizing cancer as a genetic disease (12–16). Over the past two decades, many oncogenes and tumor suppressor genes have been discovered, providing new insights into the occurrence of human tumors and growth, differentiation, and cell cycle arrest (17–19). In recent years, morbidity and mortality rates have been increasing for many cancers, such as pancreatic, lung, and colorectal cancers (20–22). Therefore, it is crucial to investigate the mechanisms underlying tumor initiation and metastasis and identify potential therapeutic targets.

While homologous box genes are typically linked to organ development, recent studies have highlighted the involvement of PITX1 in cancer development, as it has been shown to activate the transcription of p53 (23) and RASAL1 (RAS protein activator like-1) (24). The expression of PITX1 exhibits tumor-type specific variation, and it functions as a tumor suppressor gene in several human cancer types. Notably, PITX1 is frequently downregulated in malignant cancers, such as oral squamous cell carcinoma (25), malignant melanoma (26), esophageal cancer (27), lung cancer (28), colorectal cancer (29), and gastric cancer (30). However, it is highly expressed in breast cancer (31, 32), lung cancer (33, 34), prostate cancer (35), and skin squamous cell carcinoma (36) (**Table 1**), indicating that the involvement of PITX1 in the process of carcinogenesis should not be underestimated. Therefore, in this chapter, we will explain the significance of PITX1 in various tumors.

**Table 1:** The different cancer types with abnormal PITX1 expression are listed along with the corresponding prognosis.

Cancer type	PITX1 expression	Prognosis of Higher PITX1
Bladder	↓(24)	Not given
Breast	↑(31, 32)	Worse (31, 32)
Colorectal	↓(29)	Better (29)
Esophageal	↓(27, 37)	Not given
Gastric	↓(38)	Better (38)
Head and Neck	↑ (39); ↓ (40)	Better (39); Worse (40)
Kidney	↑(41); ↓ (42)	Worse (41)
Leukemia	↑ (43)	Not given
Liver	↓(44)	Not given
Lung	↑(33, 34) ↓(28, 45)	Worse (34)
Melanoma	↓ (26)	Not given
Osteosarcoma	↑ (46)	Worse (46)
Prostate	↑(35)	Worse (35)
Skin	↑(36)	Not given

The middle column contains arrows that indicate whether the expression is increased (↑) or decreased (↓).

### 2.1 Breast cancer

Among women, breast cancer (BC) is the most frequently diagnosed malignant tumor and a significant contributor to cancer-related deaths worldwide (47). Despite the effectiveness of local surgical interventions, conventional chemotherapy, radiation therapy, endocrine therapy, and monoclonal antibodies in improving the prognosis of breast cancer patients, a significant number of patients still experience the risk of recurrence and mortality. Liu et al. observed that PITX1 could directly bind to the promoter of p53, thereby transcribing and activating p53 in breast cancer cells, which in turn activates DNA damage, oncogene activation, hypoxia, nutrient deficiency, and other stress-related signaling pathways (23). For example, ionizing radiation, UV irradiation, and DNA-damaging drugs (such as 5-fluorouracil) can activate PITX1 to directly activate p53, promoting the transcriptional activation of target genes, leading to cell cycle arrest or apoptosis. According to Wang et al. (32), the expression of the PITX1 gene was found to be significantly elevated in invasive breast cancer, invasive ductal carcinoma, invasive lobular breast cancer, and ductal breast cancer compared to normal breast tissue. Additionally, PITX1 expression was observed to be elevated in patients with ER-negative and HER2-positive breast cancer, as well as in those with lymph node invasion. In terms of prognosis, higher PITX1 gene expression was positively correlated with poor overall survival, distant metastasis-free survival, recurrence-free survival, and disease-specific survival. In conclusion, PITX1 expression is upregulated in aggressive breast cancer subtypes associated with poor prognosis, including ER-negative, HER2-positive, lymph node-positive, and basal cell carcinoma. Another report showed that Erα and PITX1 directly interact with each other and inhibit the function of Erα (31). The findings indicate that PITX1 has the potential to serve as a biomarker and therapeutic target for endocrine therapy in breast cancer treatment. Downregulating PITX1 may be a possible strategy to develop new treatments for ER-positive breast cancer.

### 2.2 Colon cancer

PITX1 has been reported to be involved in tumor formation in colon cancer (48, 49). In a large-scale sample test for colon cancer in East Asian populations (2098 cases in the experimental group and 5749 cases in the control group) (50), PITX1 was identified as a new susceptibility gene that revealed a higher risk variant in East Asian populations than in European populations. A clinicopathologic study revealed that approximately 50% of colon cancer patients had reduced expression of PITX1, and a significant correlation was observed between the low expression of PITX1 and shorter prognosis, worse staging, and more positive lymph node metastasis in colon cancer patients (29). This suggests that PITX1 may act as a tumor suppressor gene and participate in the growth and metastasis of colon cancer. In addition, PITX1 is involved in multiple signaling pathways, suppressing tumorigenesis by downregulating the RAS pathway and upregulating the p53 pathway (23, 24). Frequent mutations in the RAS and p53 signaling pathways have been observed in colon tumors. Inhibition of PITX1 leads to activation of the RAS pathway and promotes tumorigenesis, whereas restoration of PITX1 in colon cancer cells suppresses tumorigenesis (Chapter 3 provides a detailed explanation of this phenomenon).

### 2.3 Esophageal squamous cell carcinoma

One of the most prevalent and deadly cancers in the world, particularly in Asian nations, is esophageal squamous cell carcinoma (ESCC), which includes adenocarcinoma and squamous cell carcinoma (51, 52). Dysregulated DNA methylation can lead to the inactivation of tumor suppressor genes and activation of oncogenes, contributing to the accumulation of genetic and epigenetic changes in the mucosa that are associated with ESCC (53, 54). Otsubo et al. conducted bisulfite sequencing analysis and confirmed that PITX1 exhibits significant hypermethylation in esophageal squamous cell carcinoma (ESCC) compared to normal tissue (*P* < 0.0001). Moreover, this hypermethylation is significantly correlated with poor prognosis in ESCC (37, 55). Besides, the expression level of PITX1 was determined in 32 paired normal and ESCC patient tissue samples, and a significant decrease in PITX1 expression was observed in tumor tissue. Moreover, overexpression of PITX1 in esophageal squamous cell carcinoma cell lines significantly inhibited tumor growth *in vivo*. The results indicate that PITX1 functions as a tumor suppressor gene in ESCC and that the hypermethylation of its promoter could serve as a potential biomarker for predicting ESCC prognosis.

### 2.4 Gastric cancer

Gastric cancer (GC) is a widespread malignancy of the digestive system with a high global incidence rate, especially prevalent in China and Japan (56, 57). Despite significant efforts in recent years, clinical treatment efficacy remains limited, with a 5-year survival rate about 30% to 50%. Surgical resection remains the preferred and key treatment strategy, and postoperative chemotherapy can effectively prevent GC metastasis and recurrence (58–60). However, chemotherapy resistance severely limits its efficacy. Therefore, it is necessary to search for new therapeutic targets to improve the poor prognosis of gastric cancer. Chen et al. evaluated the expression level of PITX1 by immunohistochemistry in 83 gastric cancer patients (29). The results showed that PITX1 was strongly or moderately expressed (100%) in the normal gastric mucosa, while 55 of 83 gastric cancer samples (66.3%) showed oppositely reduced the PITX1 expression. Notably, PITX1 expression level was significantly negative correlated with gastric cancer differentiation, size, and infiltration depth (r = -0.316, *P* < 0.01).

Another study demonstrated that decreased PITX1 expression levels in both GC tissues and GC cell lines (38). Overexpression of PITX1 significantly suppressed GC cell proliferation and tumorigenicity *in vitro* and *in vivo*, knockdown of PITX1 prevented its inhibition of GC cell proliferation, and low levels of PITX1 protein were associated with a poor prognosis in GC patients. The higher PITX1 expression suppressed cell proliferation by inducing apoptosis through targeting the apoptosis-related gene PDCD5 promoter and blocking the G1/S phase of the cell cycle *in vitro* and *in vivo*. In addition, overexpression of PITX1 increased GC cell sensitivity to 5-fluorouracil and cisplatin treatment, while silencing of PITX1 promoted GC cell resistance (61).

### 2.5 Head and neck squamous cell carcinoma

Takenobu et al. conducted immunohistochemical experiments to analyze the correlation between PITX1 expression and clinical treatment outcomes. Their findings indicated lower expression of PITX1 in HNSC. They further examined the relationship between PITX1 expression and the response to clinical treatment involving drugs like cisplatin and 5-fluorouracil. Their analysis revealed that patients with lower PITX1 expression showed more progressive disease (PD) and stable disease (SD) compared to those with complete response (CR), suggesting that higher PITX1 expression in HNSC is associated with increased chemotherapy sensitivity. Consequently, patients with higher PITX1 expression exhibited improved prognoses, aligning with their chemotherapy response (39).

Oddly, an opposing result was reported by Zhao and Libório et al. Using the MethSurv and UCSC Xena databases, they predicted higher methylation levels of PITX1 in HNSC than in normal tissues, leading to a significant reduction in PITX1 expression. This reduction might be linked to PITX1 hypermethylation. Additionally, patients with elevated PITX1 expression experienced worse stage, grade, and relapse-free survival (RFS) which was an opposing result of Takenobu et al. The databases also suggested correlations between PITX1 and immune cell expression, including CD4+ and CD8+ T cells (25, 40). However, relying solely on public databases might limit the accuracy of these predictions. Further cell biology verification is essential to provide clearer insights into the functional role of PITX1 and its correlations.

### 2.6 Hepatocellular carcinoma

The worldwide incidence of hepatocellular carcinoma (HCC) ranks fifth among all cancers and is the second leading cause of cancer-related mortality. HCC is the predominant primary liver cancer, accounting for 75-85% of cases, followed by intrahepatic bile duct carcinoma, which accounts for 10-15% of cases, and other rare types (62). The incidence and mortality rates of HCC remain poor, and the 5-year survival rate for late-stage HCC is less than 5% (63). These dismal outcomes are partly due to insufficient biomarkers for timely diagnosis in HCC, which contributes to the unsatisfactory prognosis, with only 30-40% of patients being diagnosed and treated with potentially curative therapies (64). Currently, alpha-fetoprotein (AFP) and abdominal liver ultrasound are the most widely used methods for HCC identification. Liver ultrasound is undoubtedly an effective method for detecting HCC, with both sensitivity and specificity of 80-95% (65). However, imaging technology improvements can detect nodular lesions smaller than 1 cm, repeated liver ultrasound and other imaging methods are economically burdensome (66). Eun et al. tested the diagnostic ability of PITX1 for liver cancer (n = 63) and compared it with the AFP group (44). The results showed that PITX1 (AUC = 0.889) was more sensitive and specific (*P* < 0.0001) than AFP (AUC = 0.767). The experiment also found that both PITX1 mRNA and protein expression in adjacent tissues, well-differentiated HCC tissues, and poor-differentiated HCC tissues decreased significantly in a stepwise manner, and the expression level of PITX1 in liver cancer tissue of patients with serum AFP > 400 μg/L was significantly lower than that in patients with AFP < 400 μg/L. This finding indicates that PITX1 exhibits tumor biomarker properties and shows reduced expression in poorly differentiated liver cancer tissue.

### 2.7 Kidney renal clear cell carcinoma

Within the context of normal proximal tubular cells in the kidney, the direct interaction of PITX1 with the promoter region of melatonin receptor 1A (MTNR1A) serves as a pivotal mechanism for fostering MTNR1A expression. However, in the event of kidney injury, the diminished aggregation of PITX1 precipitates a corresponding reduction in MTNR1A expression levels. The employment of the MTNR1A antagonist, Luzindole, subsequently triggers the attenuation of CDH1, Per2, and αSMA expression. This cascade of events culminates in the escalation of renal injury severity. The implications drawn from these findings underscore the role of PITX1 in preserving the normative function of the kidney via MTNR1A, thus conferring a protective effect against the onset of renal failure (67).

In a study focused on KIRC, miR-886-3p demonstrated heightened expression, while PITX1 appeared to be underexpressed as a tumor suppressor gene. Suppression of miR-886-3p led to inhibited cell proliferation and facilitated apoptosis. This miRNA interacts with the CACCCGC site on the 3’-UTR of PITX1, causing PITX1 expression repression. Additionally, PITX1 downregulation was identified in 90% (18/20) of KIRC patient tissues (42). Curiously, an alternate investigation analyzed PITX1 expression in KIRC through TCGA, TIMER, and GEO databases, finding high mRNA expression levels in tumor tissues alongside hypermethylated PITX1 DNA. This was further validated by qRT-PCR in clinical samples (n = 10), consistently highlighting high PITX1 expression’s positive correlation with KIRC stage, grade, prognosis, and metastasis in various databases. This cumulative evidence proposes that elevated PITX1 expression independently predicts poor prognosis risk in KIRC (41, 68). The contrasting findings between the earlier and later research possibly stem from the limited number of clinical tissues studied initially. Furthermore, the involved signaling pathway in KIRC remains undisclosed. Future investigations should delve into PITX1’s role within KIRC, particularly with regard to its regulatory mechanisms and signaling pathways.

### 2.8 Leukemia

PITX1 exhibits heightened expression in 9% of leukemia cases (43). While elevated PITX1 expression does not significantly impact proliferation in the leukemia cell line JURKAT, it does contribute to the upregulation of genes associated with T cell differentiation. For instance, PITX1 interacts with the 3473 bp promoter region of RUNX1, thereby repressing RUNX1 expression and consequently fostering the expression of CD4+ and facilitating T cell differentiation. Moreover, PITX1 also forms associations with the target gene TRIB2 within the NOTCH signaling pathway.

### 2.9 Lung cancer

Lung cancer is one of the most common types of cancer and a leading cause of cancer-related deaths. In the United States alone, it is estimated that nearly 60,000 people die from lung cancer each year and over 11,000 new cases are diagnosed annually (22). Despite significant advances in targeted therapy and immunotherapy over the past few decades, the five-year survival rate for lung cancer patients remains low. The spread of tumor cells from the primary site to the bones through the bloodstream or lymphatic system is a common complication in advanced lung cancer. Bone metastasis can severely damage bone tissue and even lead to pathological fractures. An animal study has shown that high levels of PITX1 significantly increase the bone metastatic activity of lung cancer cells, stimulating increased osteoclast and proteolytic activity and promoting aggressive bone damage (33). Some studies have reported a relationship between DNA methylation of the PITX family and survival. For example, according to Song et al. (34), PITX1 was found to be overexpressed in lung cancer tissue based on bioinformatics analysis. Moreover, high levels of methylation of the PITX1 promoter were found to be correlated with poorer overall survival in lung cancer patients. Strangely, another independent experimental study showed that PITX1 was reduced in lung cancer tissue compared to normal lung tissue (28). This observation contradicts the findings reported by Song et al., possibly attributed to the utilization of public databases for data retrieval rather than experimental research. Consequently, additional investigation is warranted to assess the reliability of these data sources.

### 2.10 Melanoma

A study employed immunohistochemical and immunofluorescence methods to assess PITX1 expression in normal skin and melanoma tissue. The study revealed a marked reduction in PITX1 expression within melanomas, which was also correlated with melanoma staging (26). Another investigation reported that PITX1’s homologous structure interacts with ZCCHC10’s CCHC domain, forming a complex that directly binds to the hTERT promoter region, resulting in transcriptional silencing of the telomerase reverse transcriptase hTERT. Overexpression of PITX1 and ZCCHC10 inhibited hTERT expression, thus suppressing melanoma proliferation (69). Additionally, microRNA-19b (miR-19b) has been identified to regulate hTERT expression and cell proliferation by binding to the 3’UTR region of PITX1 at position 912-919. In melanoma, miR-19b is highly expressed, leading to decreased PITX1 expression compared to normal skin tissue. Overexpressing miR-19b in melanoma cell lines (A2058) significantly suppressed PITX1 expression, resulting in a 1.5 to 1.7-fold increase in telomerase activity and enhanced melanoma cell proliferation. Conversely, knocking down miR-19b yielded the opposite results (70). Another report revealed that both PITX1 and SOX9 expressions were reduced in melanoma. PITX1 overexpression facilitated SOX9 expression by binding to the RE1 (-592/-588) and RE3 (-520/-504) sites within the SOX9 promoter region, thereby inhibiting melanoma cell proliferation and promoting apoptosis.

### 2.11 Osteosarcoma

Zhang et al. conducted an immunohistochemical analysis of PITX1 expression in six cases of normal lower limb bone tissues and thirty-five cases of osteosarcoma. Their findings indicated significant upregulation of PITX1 in osteosarcoma, exhibiting a negative correlation with prognosis and lung metastasis (46). Moreover, heightened PITX1 expression correlated with improved outcomes in osteosarcoma (71), contributing to tumor inhibition via the modulation of Wnt/β-catenin, SMD, and Hippo signaling pathways. PITX1 directly interacts with STAT3, resulting in reduced STAT3 activity and consequent inhibition of the transcriptional activation of LINC00662. This inhibition of LINC00662, in turn, suppresses the proliferation and metastasis of osteosarcoma cells (further details to be elaborated in subsequent sections).

### 2.12 Ovarian cancer

Analogous to the observations in breast cancer, the interaction between PITX1 and ERα has been substantiated in ovarian cancer cell lines (72). While ERα’s pivotal engagement in the PI3K/AKT and RAS/ERK signaling pathways within the context of ovarian cancer has undergone extensive elucidation, the present study refrained from delving into the specific functions of PITX1 and ERα in this ovarian cancer context (73). Thus, there exists a compelling rationale for conducting further in-depth investigations. Furthermore, it has come to light that PITX1 operates as a key regulator of BMP15 expression by virtue of its binding to the promoter region (74). PITX1 and BMP15 demonstrate consistent patterns of expression in mouse ovarian cells. Regrettably, despite the documented implication of PITX1 in hormone synthesis, the realm of hormone secretion within the ovary and its potential role in conferring resistance to hormone therapy in ovarian cancer remain shrouded in limited information. This conspicuous void underscores the imperative of embarking upon comprehensive exploration into the multifaceted role that PITX1 plays in the context of ovarian cancer.

### 2.13 Prostate cancer

In prostate cancer patient tissues, about two-thirds of tumor tissues overexpress PITX1, which is associated with higher tumor invasiveness characteristics such as advanced tumor stage, higher Gleason score, more lymph node metastases, positive surgical margins, and higher Ki67 index, indicating a worse prognosis (35). One hallmark of cancer is telomere maintenance, and telomerase is a ribonucleoprotein enzyme that maintains telomere length through reverse transcription and is crucial for cellular immortalization and cancer progression (75–77). Telomerase activity is primarily attributed to the existence of telomerase reverse transcriptase (TERT) (78). Poos et al. conducted *in vitro* and *in vivo* experiments that confirmed PITX1 as a positive transcriptional regulator, which binds to the TERT promoter to modulate telomerase activity (35). The research showed a positive correlation between PITX1 gene expression and telomere length, which prolonged cell lifespan and facilitated telomerase ribonucleic acid enzyme-mediated extension of the telomere repeat sequence at chromosome ends, which helps to maintain cell proliferation. Oppositely, knockdown of PITX1 in prostate cancer cell lines reduces TERT expression. Interestingly, in contrast to the findings of Poos et al., an independent study reported a direct interaction between the homologous domain of PITX1 and the CCHC domain of ZCCHC10 with the TERT promoter region, resulting in the inhibition of TERT function, transcription, and cell growth (69), which contradicted the results of Poos et al.’s study. Perhaps the function of PITX1 in regulating telomeres is quite complicated and requires further investigation.

## 3 PITX1-mediated cell apoptosis

The tumor suppressor gene p53 promoter can be directly bound by PITX1, which in turn increases the expression of p53 (23). After 24 hours, cells that overexpressed PITX1 exhibited 43% apoptosis, compared to a proportion of 9% in cells transfected with the vector. In contrast, the reduction of p53 expression by siRNA led to a significant decrease in PITX1 expression levels, resulting in a threefold decrease in induced cell apoptosis (46% vs. 14%). However, when the author used another osteosarcoma cell line MG-63 (lacking the p53 gene), overexpression of PITX1 still induced cell apoptosis, suggesting that PITX1 may promote cell apoptosis through a non-p53-dependent pathway.

**Figure 2:**
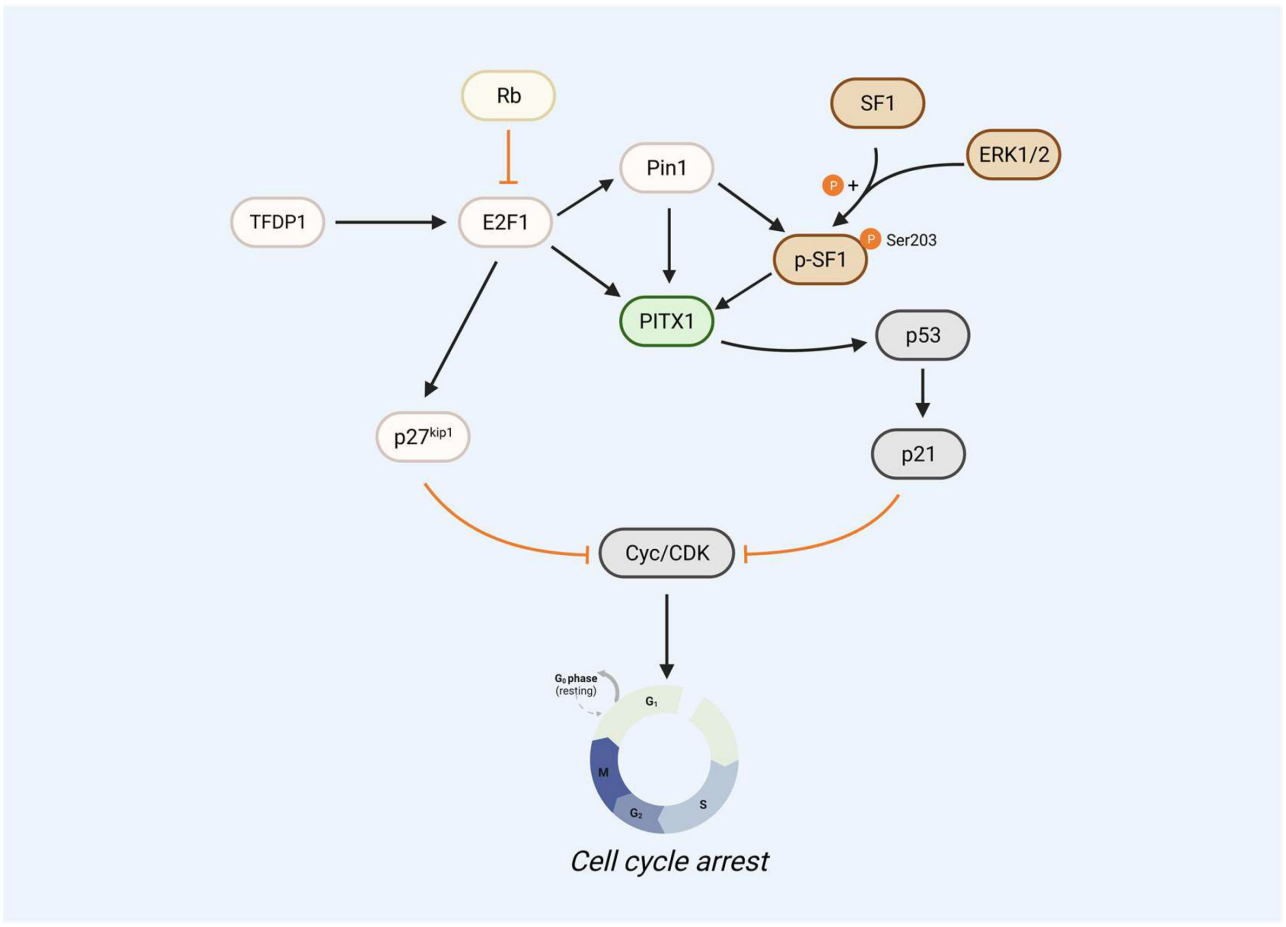
PITX1 acts as a core gene that regulates the p53 signaling pathway. E2F1 as upstream of PITX1 can upregulate PITX1 expression levels by directly binding to its proximal promoter region, thereby promoting the PITX1-p53-p21 signaling pathway to cause cell cycle arrest. The mutation/deletion of Rb determines the stimulation of proliferative gene E2F1, including the ability to activate p27^kip1^ and Pin1, which can directly inhibit the translation of cyclin E and CDK2, leading to cell cycle arrest. Pin1 can bind PITX1 to phosphorylated SF1, and SF-1 S203 site phosphorylation cooperates with Pin1 to increase SF-1-PITX1 interactions to promote downstream genes, but SF-1 S203 site phosphorylation is regulated by ERK1/2.

E2F1 is a member also involved in cell apoptosis. E2F1 can upregulate PITX1 expression by directly binding to its proximal promoter domain, thereby promoting cell cycle arrest (2). Since TFDP1 is the primary heterodimeric partner of E2F1 (79), TFDP1 also regulates the PITX1 gene. Reduced the expression of TFDP1 in human chondrocytes decreased PITX1 at both mRNA and protein levels. Luciferase assays showed that low TFDP1 levels also reduced E2F1 activity. TFDP1 can form a dimer with E2F1 to act as a transcriptional regulator, which then binds to the PITX1 promoter region to upregulate PITX1 expression, promoting the activation of the p53 pathway (which was mentioned above). In addition, several studies have also shown that the expression of p27^kip1^ and Pin1 is mediated and the activated by of E2F1 (80–83), and the mutation/deletion of Rb determines the stimulation of proliferative genes by E2F1 and activates p27^kip1^ and Pin1. The cooperative phosphorylation of SF-1 at the S203 site with Pin1 enhances the interaction between SF-1 and PITX1, thereby promoting downstream gene expression. Meanwhile, Pin1 can also directly bind to PITX1 (S207A, S259A sites) to promote PITX1 stability and inhibit PITX1 degradation via the proteasome pathway, leading to high expression of PITX1 and suppression of the cell cycle regulator cyclin D1 (**Figure 2**).

## 4 Post-transcriptional modifications of PITX1

### 4.1 PITX1 phosphorylation

**Figure 3:**
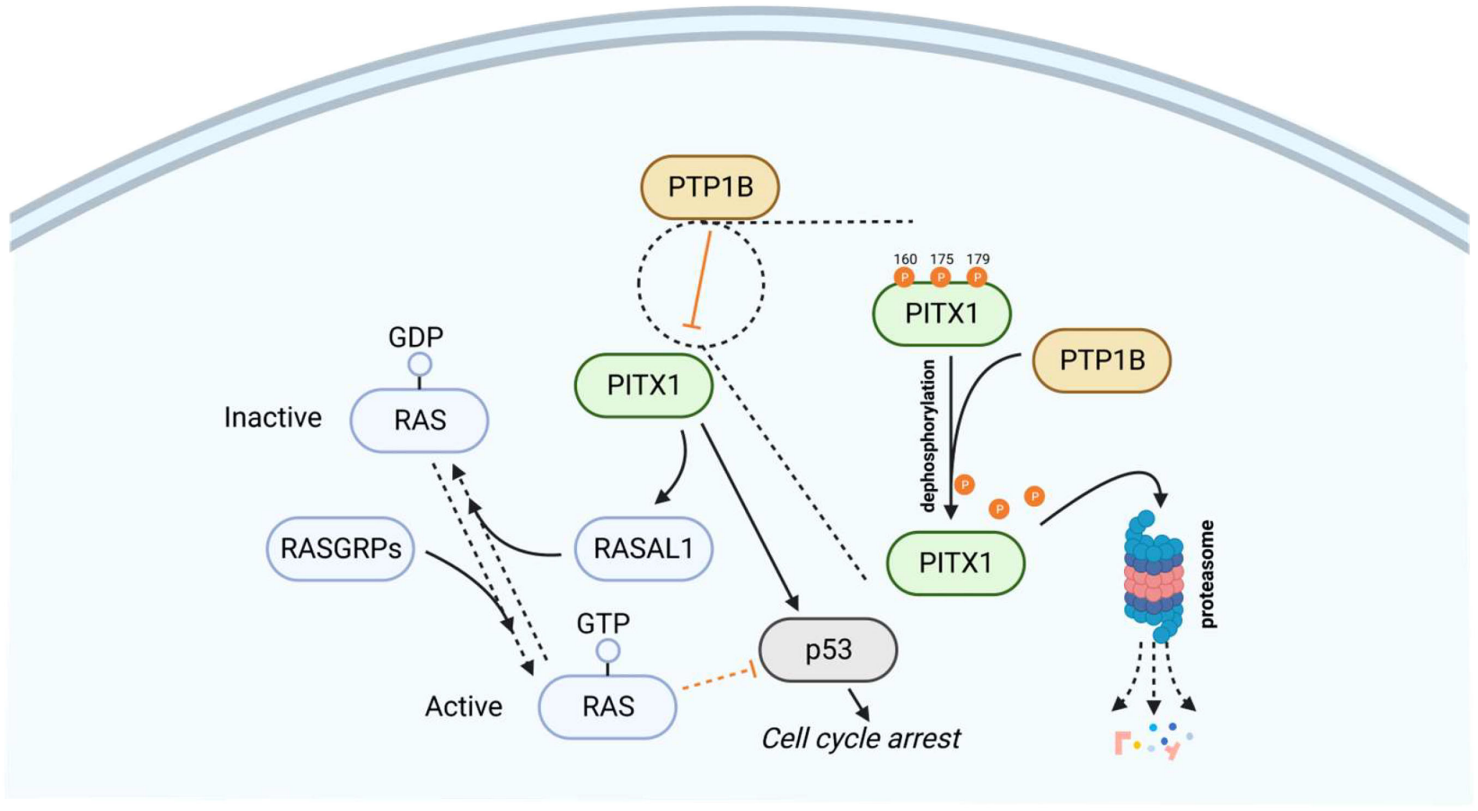
PTP1B dephosphorylates and promotes the degradation of PITX1 via the proteasome pathway. As upstream of PITX1, PTP1B is directly involved in inhibiting the expression of PITX1, as shown in the magnification of the dashed circle area, PTP1B can dephosphorylate the 160, 175 and 179 sites of PITX1, making PITX1 unstable and degraded through the proteasome pathway, so that PITX1 cannot promote RASAL1-induced RAS inactivation and initiation of the p53 signaling pathway. Therefore, overexpression of PTP1B can inhibit PITX1-mediated cell cycle arrest and promote tumor cell survival.

Protein tyrosine phosphatase 1B (PTP1B) directly dephosphorylates PITX1 at Y160, Y175, and Y179 sites, thereby further weakening the protein stability of PITX1, promoting the PITX1 proteasome degradation pathway. Furthermore, single-size mutations of Y160, Y175, and Y179 in PITX1 can block the effect of PTP1B-mediated dephosphorylation. In addition, the dephosphorylation process of PTP1B can be blocked by sorafenib or regorafenib. Sorafenib is the first targeted drug approved for hepatocellular carcinoma, which can directly interact with PTP1B to reduce its activity, causing excessive phosphorylation of PITX1, promoting PITX1 stability, and promoting the expression of PITX1 and RASAL1, thereby inducing apoptosis of tumor cells and inactivation of the RAS gene, resulting in the promotion of tumor cell apoptosis by sorafenib (**Figure 3**). Overexpression of PTP1B can weaken the efficacy of sorafenib and regorafenib in cancer cells, and this PTP1B-dependent PITX-RASAL1-RAS axis change also directly affects the efficacy of sorafenib in treating liver cancer (84, 85). In the realm of PITX1-mediated apoptosis, a significant role for RAS emerges. The phenomenon of both RAS oncogene overexpression and PITX1 knockdown within tumors yielding analogous cancer-promoting traits (such as proliferation, invasion, and migration) highlights the potential significance of RAS in this context. This suggests that PITX1 potentially exerts a negative regulatory influence on the RAS signaling pathway. To delve deeper, PITX1 is observed to bind to the RASAL1 transcription start site, TAAGCC, thereby facilitating the transcriptional enhancement of RASAL1. Consequently, by orchestrating Ca2+ signal transmission and the hydrolysis of RAS-GTP, PITX1 contributes to RAS gene inactivation, curbing the state of RAS-GTP activation. Consequently, this quelling of RAS-GTP activity inhibits the activation of the RAS/ERK signaling pathway and hampers tumor proliferation (24, 86–88). In a parallel facet, an additional player, p120RasGAP, also features in the modulation of RAS activity. Like RASAL1, p120RasGAP is associated with attenuating GTPase function and promotes the inactive GDP-bound form of RAS. Overexpressing PTP1B curtails the expression of both p120RasGAP and PITX1 in hepatocellular carcinoma (HCC) cells, resulting in inhibition of the PITX1-p120RasGAP axis. This axis suppression augments RAS gene expression, consequently fostering tumor cell proliferation (84). Intriguingly, a curious assertion by the author posits that PITX1 directly activates p120RasGAP through transcription to influence subsequent signaling pathways. However, empirical evidence validating this claim is conspicuously absent. At present, there exists no experimental validation of an interaction between PITX1 and p120RasGAP. Consequently, the precise manner in which PITX1, either directly or indirectly, modulates p120RasGAP expression, and its ramifications for reduced RAS activity necessitate further elucidation and reporting.

In another report (89), PITX1 can be phosphorylated by c-Abl, which leads to cell apoptosis. STI571, a c-Abl kinase inhibitor, can prevent Adriamycin-induced expression of PITX1. c-Abl-mediated phosphorylation of PITX1 stabilizes the protein and facilitates the apoptotic pathway. Although the specific interaction site of PITX1 was not mentioned in this article, and the downstream pathway was not fully elucidated, it is undeniable that PITX1 phosphorylation participates in important cell apoptosis-related pathways.

### 4.2 PITX1 and DNA methylation

DNA methylation is a chemical modification that regulates epigenetic gene expression (90, 91). According to several studies, the DNA methylation and survival of the PITX gene family are related. For instance, Sailer et al. indicated that the expression of PITX3 DNA methylation was associated with overall survival in head and neck squamous cell carcinoma (92). Clinical validation of PITX2 DNA methylation can serve as an outstanding biomarker for predicting the chemotherapy resistance (93, 94). In addition, in Song’s study (34), they examined the prognostic significance of the PITX gene family and PITX DNA methylation levels in 516 patients with lung adenocarcinoma (LUAD) using data from public databases. Elevated PITX1 DNA methylation levels were found to be associated with poor overall survival (OS) in LUAD patients. Another study also demonstrated that PITX1 was highly methylated (*P* < 0.0001) in 40 surgically resected esophageal squamous cell carcinoma tissues (37), which was related to cancer infiltrating depth (*P* = 0.0011), cancer stage (*P* = 0.0052), and associated with poor survival in ESCC (hazard ratio (HR): 0.1538; 95% confidence interval (95% CI: 0.03159-0.7488; *P* = 0.0169). Furthermore, it is noteworthy that in patients with head and neck squamous cell carcinoma, the extent of PITX1 exon 3 methylation within cancerous tissue was markedly elevated in comparison to adjacent normal tissue (PITX1 exon 3 methylation: tumor tissue 58.1%; adjacent tissue 31.7%, *P* < 0.001). Nevertheless, it is imperative to highlight that these studies did not proceed to delve into the precise molecular entities that interplay with PITX1, thereby inducing its heightened methylation status. Consequently, a comprehensive understanding of the intricate signaling pathways involved in this process remains an area necessitating further refinement and exploration.

## 5 PITX1 and noncoding RNA

**Figure 4:**
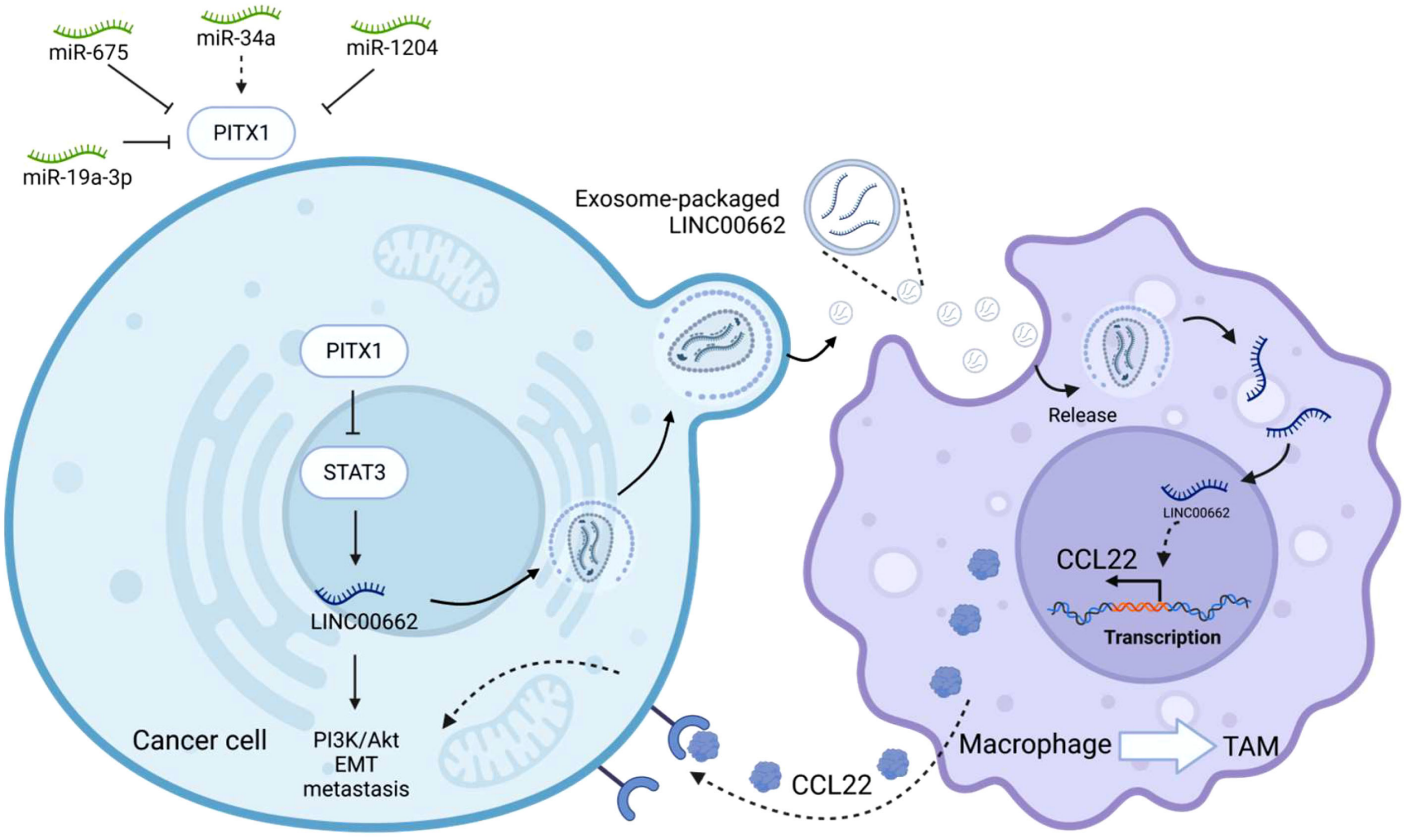
PITX1 regulates noncoding RNAs. PITX1, as the upstream of STAT3, can directly inhibit the transcriptional activity of STAT3, and then inhibit LINC00662-mediated EMT and metastasis in tumor cells. LINC00662 can be encapsulated by exosomes and then secreted extracellularly to be captured by macrophages, LINC00662 can promote the transformation of macrophages into tumor-associated macrophages (TAM), and promote the transcription and translation of inflammatory factor CCL22 in macrophages, macrophages secrete CCL22 outside the cell to be received by tumor cells, thereby promoting the proliferation and metastasis of tumor cells, and overexpression of PITX1 can inhibit this process. In the upper left corner of the image are other noncoding RNAs that can be involved in regulating PITX1.

LINC00662 is located on chromosome 19q11 and has a full length of 2085 bp. Increased expression of LINC00662 is intimately associated with cancer cell growth, invasion, migration, apoptosis, cell cycle, EMT and prognosis (95). It promotes tumor progression by regulating the Wnt/β-catenin, SMD, and Hippo signaling pathways. PITX1 has been implicated in the regulation of LINC00662 expression by directly binding to STAT3 and suppressing transcriptional activation of LINC00662 (71). ChIP and luciferase assay analysis showed that STAT3 occupied the -1992 ~ -1982 and -1875 ~ -1865 sites in the LINC00662 promoter, thereby promoting transcriptional activation of LINC00662. It is involved in the PI3k/Akt signaling pathway. Furthermore, overexpression of PITX1 reduces the expression level of STAT3, promotes its degradation through the proteasome pathway, inhibits the expression of LINC00662, and thus suppresses EMT and tumor proliferation. Elevated PITX1 inhibits osteosarcoma (OS) cell proliferation and migration. Nude mice injected with tumor cells via tail intravenous injection to simulate tumor cell metastasis, showed a significant reduction in the number and size of lung metastases with the overexpression of PITX1 in tumor cells. Regarding the expression of EMT biomarkers, it was found that elevated PITX1 significantly increased the expression of E-cadherin and decreased the expression of β-catenin and vimentin in OS cells, indicating that PITX1 inhibits tumor cell adhesion and EMT. Interestingly, exosome-packaged LINC00662 can serve as a “seed” and be transferred to macrophages. Co-culture with macrophages and exosome-encapsulated LINC00662 promotes M2 polarization, leading to their recruitment as tumor-associated macrophages (TAMs), which in turn promotes the expression of the secretory inflammatory cytokine CCL22 (**Figure 4**). In addition, treating osteosarcoma cells with the inflammatory factor CCL22 was found to promote EMT transformation, enhance cell invasion and migration, and promote metastasis. Notably, treatment with a CCL22 antibody, exosome inhibitor GW4869 or simultaneous overexpression of PITX1 can block these effects. Meanwhile, PITX1 has been reported to be involved in interferon synthesis (96). After viral induction, the homeodomain (HD) of PITX1 specifically recognizes the distal negative regulatory element (DNRE) in the IFN-A11 (Interferon A11) promoter and transcribes it. When PITX1 is overexpressed in cells, it significantly inhibits the expression of IFN-A11 by negatively regulating the DNRE-containing IFN-A11 promoter. This may suggest that PITX1 is not only a transcriptional activator but also a suppressor of gene expression.

Furthermore, Liu and Qiao demonstrated through a dual-luciferase reporter assay in gastric cancer cell lines that PITX1 targets miR-675 and miR-19a-3p and promotes tumor growth through activating of Wnt/β-catenin and PDCD5 (38, 97). Overexpression of PITX1 reversed the effects of miR-675 on EMT markers and positively regulated gastric cancer proliferation and invasion through the EMT and WNT/β-catenin signaling pathways, indicating that the miR-675/PITX1 axis may regulate GC invasiveness by modulating EMT. Additionally, another microRNA, miR-19a-3p, can inhibit PITX1 transcription and translation by specifically binding to PITX1 mRNA sequence.

miR-1204 is a member of the PVT1 region located on human chromosome 8q24. The expression of miR-1204 is significantly positively correlated with NSCLC tumor size, lymph node metastasis, and TNM stage (45). NSCLC cell line tumor progression is associated with an upregulation of miR-1204, which may be attributed to its direct binding to the 3’-UTR of PITX1, resulting in downregulated PITX1 expression and enhanced proliferation of NSCLC cells.

Other reports showed that miR-34a can indirectly affect E2F1, thereby inducing PITX1-mediated apoptosis (98). Curcumin can significantly upregulate E2F1/PITX1 and decrease cell apoptosis by inhibiting miR-34a, as well as possibly enhancing autophagy through the Akt/mTOR pathway. The anti-apoptotic effect of curcumin may involve downregulation of miR-34a and upregulation of E2F1 and PITX1 expression. Agomir-34a significantly decreased the expression of E2F1 and PITX1 in rats treated with low-dose curcumin, but not in those treated with high-dose curcumin. TUNEL staining analysis showed that high-dose curcumin had a more significant inhibitory effect on miR-34a expression and promoted cancer cell proliferation. This article suggests that PITX1 may be an anti-apoptotic gene, and its overexpression can inhibit tumor cell apoptosis. However, the authors did not specify the mechanism by which PITX1 induces this abnormal proliferation function, as most studies have identified PITX1 acted as a tumor suppressor gene. Further investigation may be required to clarify this.

Circular RNAs (circRNAs) have garnered recognition as prominent contributors to human malignancies, with a multitude of circRNAs identified to be engaged in pivotal signaling pathways within tumors (99–101). Among these, circ-PITX1 (hsa_circ_0074027) stands out, arising from the PITX1 locus on chr5: 134363423–134369964. Originally documented by Salzman et al. in A549 cells, circPITX1’s emergence has been associated with potential implications (102–104). Notably, it exhibits heightened expression in glioma tissues and cell lines, showcasing a positive correlation with tumor size and staging. The functional perturbation of circPITX1 through knockdown within glioma cell lines manifests a remarkable array of effects—namely, proliferation inhibition, glycolysis modulation, enhanced radiosensitivity, and a notable attenuation of tumor growth *in vivo*. CircPITX1 orchestrates its actions through multifaceted mechanisms. It triggers the upregulation of NIMA-related kinase 2 (NEK2) expression, intensifying glycolytic processes via intricate interactions involving the GGUGUGU motif and miR-329-3p sequestration. This orchestration consequently underscores the promotion of tumor growth and the fortification of radiotherapy resistance (105, 106). Moreover, circPITX1’s influence extends to miR-584-5p, acting as a molecular sponge that hinders its regulatory activity. Of particular interest is miR-584-5p’s capacity to suppress KPNB1, consequently affecting the modulation exerted by KPNB1 on glioma proliferation and cell cycle progression. It is noteworthy that circPITX1’s role as a miR-584-5p sponge substantiates its involvement in regulating KPNB1 expression in glioblastoma (GBM) cells. This intricate web of interactions culminates in a consequential cascade—circPITX1’s suppression of miR-584-5p abrogates the miRNA’s inhibitory influence on KPNB1, thus ultimately culminating in the heightened expression of KPNB1 protein. This orchestrated interplay subsequently translates into augmented glioma proliferation both *in vitro* and *in vivo*, encapsulating the multifaceted role of circPITX1 in glioma progression (107).

The impact of circPITX1 is not confined to a single cancer type; its influence spans across various malignancies, including gastric cancer, non-small cell lung cancer (NSCLC), and others. In gastric cancer, circPITX1 emerges as a driver of tumor proliferation by exerting its regulatory effect on EIF4A3—a factor crucial for tumor growth (108). In the context of NSCLC, the versatile role of circPITX1 comes to the fore (109, 110). Elevated expression of circPITX1 characterizes NSCLC tissues and cells, with a noteworthy correlation with disease stage, grade, and prognosis. Acting predominantly within the cytoplasmic domain, circPITX1 leverages miR-362-3p sponging to augment clathrin heavy chain (CLTC) expression, thereby promoting NSCLC cell proliferation. Further intricacies arise as circPITX1’s intricate interaction with miR-335-5p unfolds, impacting cell progression via CULAB modulation (111). The collaborative impact of circPITX1, miR-335-5p, and CULAB on cell proliferation and apoptosis underscores the network’s significance. Subsequent scrutiny reveals circPITX1’s orchestration of RHOA-mediated processes, encompassing cytoskeletal remodeling and cell migration—a central feature in cancer metastasis (112). Delving deeper, circPITX1 demonstrates a capacity to modulate various targets, each conferring distinctive facets of NSCLC progression. Direct interaction with miR-185-3p boosts BRD4 and MADD expression via post-transcriptional enhancement, thereby invigorating tumor development (109). The miR-30e-5p/ITGA6 axis emerges as an avenue through which circPITX1 drives epithelial-mesenchymal transition (EMT) and proliferation, further fueling NSCLC progression (104). circPITX1’s complex relationship with miR-379-5p underscores its role in circumventing MAP3K2 inhibition, ultimately promoting anti-apoptotic pathways (103). Similarly, the interplay of circ-PITX1/miR-518a-5p/IL17RD and circ-PITX1/miR-1248/CCND2 signaling axes outlines an intricate network of regulatory elements (105, 113). However, it’s noteworthy that existing explorations into circPITX1 remain somewhat preliminary. While they have pinpointed target proteins engaged by circPITX1, the subsequent signaling cascades and potential therapeutic interventions are yet to be elucidated. This highlights the ongoing avenues of inquiry, urging comprehensive investigations to unravel the complete landscape of circPITX1’s impact and therapeutic potential in cancer.

## 6 PITX1 and drug resistance

Yamaguchi et al. treated tumor cells with DNA damaging agents, such as mitomycin C and etoposide, resulting in a significant increase in PITX1 expression levels (89). In addition, the study demonstrated that overexpression of PITX1 in tumor cells promoted non-p53-dependent apoptosis upon treatment with DNA damaging agents. Mechanistically, c-Abl can bind and phosphorylate PITX1 *in vitro*, promoting PITX1 stability and apoptosis. Knocking out c-Abl prevented the upregulation of PITX1 protein expression and drug resistance after treatment with DNA damaging agents. Similarly, when the c-Abl inhibitor STI571 was used, etoposide-induced apoptosis was significantly suppressed. However, due to limitations in antibody availability, the article did not identify the specific site at which c-Abl phosphorylates PITX1.

Sorafenib and regorafenib, both recognized as vascular endothelial growth factor receptor (VEGFR)-targeted drugs for advanced hepatocellular carcinoma (HCC) or colorectal cancer (CRC) patients, have exhibited the capacity to enhance the expression of PITX1 protein. This phenomenon has been expounded upon in preceding sections of this study. Specifically, the action of sorafenib or regorafenib is rooted in their direct interaction with the helix α6 and α3 regions within the catalytic domain of PTP1B. This interaction subsequently leads to a dose-dependent inhibition of PTP1B phosphorylation, thereby curtailing its enzymatic activity. This attenuation of PTP1B activity translates to a reduction in the phosphorylation of PITX1 at critical Y160, Y175, and Y179 sites. This pivotal inhibition of PITX1 phosphorylation curtails its ubiquitin-proteasome pathway-mediated degradation, engendering heightened stability within the PITX1-RASAL1-RAS signaling cascade. Ultimately, this orchestrated series of events culminates in the imposition of a non-activated state upon the RAS oncogene—a pivotal shift contributing to the augmentation of tumor cell apoptosis (84, 85).

In several separate studies, overexpression of PITX1 participated in muscular dystrophy regulatory pathway involving DUX4 and p53 (114–116), PITX1 expression was inhibited by morpholino (a drug that blocks the translation of macromolecules with mRNA by pairing with target mRNA bases in a complementary manner), and the treatment resulting effective in improving muscle strength in transgenic mice that overexpress PITX1, indicating PITX1 as a potential therapeutic approach for muscle atrophy. However, these articles did not specify the exact binding site of morpholino or whether it directly interacts with PITX1. Additionally, another study found that overexpression of PITX1 increased the sensitivity of gastric cancer (GC) cells to 5-fluorouracil and cisplatin treatment, while silencing PITX1 promoted drug resistance in GC cells (61). The results indicate that drug treatment upregulates the expression of PITX1, and silencing PITX1 enhances drug resistance. However, the studies did not identify which particular drug can interact with PITX1. Given PITX1 potent tumor-suppressive function, future investigations into drugs that inhibit PITX1 expression may be warranted.

## 7 PITX1 and other signal pathways

The autophagy pathway is also regulated by PITX1. Specifically, upregulation of PITX1 leads to a decrease in mTOR and p-P70S6K expression, an increase in Beclin1 levels, and an enhancement of autophagy. Flow cytometry showed that cell senescence was reduced, and PITX1 overexpression reversed cell senescence by inhibiting mTOR and increasing autophagy (117). However, the article did not specify which mTORC1 or mTORC2 complexes played a dominant role, nor discussed the extent of autophagic flux. In another article, Mudie et al. demonstrated that depletion of PITX1 in HeLa cells led to a high level of p62 (an essential autophagy protein typically used as a marker of the process) and inhibited HeLa cell proliferation while promoting apoptosis, but there were no significant changes in LC3-I and LC3-II levels (118). In the hypoxia-related pathway, a decrease in PITX1 leads to a decrease in JMJD2B (a histone demethylase) expression and an increase in PARP levels, resulting in increased apoptosis. Furthermore, under hypoxic conditions, PITX1 interacts with HIF-1β to regulate HIF-1α activity to promote increased HIF-1α transcriptional activity. These results indicate that PITX1 may play a crucial role in cell survival and proliferation under hypoxic conditions, and depletion of PITX1 may induce autophagy and apoptosis in tumor cells.

PITX1 also drives astrocyte differentiation by activating the SOX9 promoter through targeting the CAATCC-binding motif region, and acts as a co-transcription factor to maintain SOX9 expression (119, 120). Knockdown experiments revealed that suppression of PITX1 resulted in decreased SOX9 expression, whereas overexpression of PITX1 led to activated and increased SOX9 expression, inhibiting the growth and proliferation of melanoma by suppressing SOX10 and SAMMSON.

PITX1 has also been reported in the growth and development of animals. In E9.5 mouse embryos, PITX1 is strongly expressed in the proximal mesenchyme of the mouse’s mandible and in the oral epithelial cells. In PITX1^-/-^ transgenic mice, delayed tooth development and a large number of apoptotic cells were observed in the mandibular alveolar bone, and the Tbx1 gene (which has been reported to play an important role in tissue development and morphogenesis) was absent from the teeth (121). In addition, PITX1^-/-^ transgenic mice exhibited early postnatal lethality, loss of ilium, patella and mandibular, decreased femur length, and deformities (8, 11), but PITX1^+/-^ mice appeared to be normal, with only mild morphological defects observed at low penetrance levels (122). These findings suggest that the loss of PITX1 may lead to cell apoptosis, and its presence may promote cell proliferation and maintain the generation of stem cells. Unfortunately, animal models have not yet been used to investigate the role of PITX1 in tumorigenesis, and it remains unknown whether PITX1 deficiency in mice will promote or inhibit tumor growth. Further studies are needed to address this question.

## 8 PITX1 also appears to play a role as an oncogene

While in most studies, PITX1 has been widely reported as a tumor suppressor gene, some researchers believe that PITX1 is an oncogenic gene that promotes the development and progression of tumors, such as the role of PITX1 in KRAS-mutant colorectal cancer cells and acute leukemia (43, 118, 123), here we summarize some of the target proteins of PITX1 and the corresponding functions (**Table 2**).

**Table 2:** Interacting partners of PITX1 and their effect on function.

Protein interactor	Outcome	References
bHLH	The interaction between bHLH factors and PITX1 promotes insulin synthesis.	(124, 125)
BMP15	PITX1 has been identified as a regulator of BMP15 expression by binding to its promoter region.	(74)
c-Abl	Under DNA damage drug treatment, c-Abl phosphorylates PITX1, promoting cell apoptosis.	(89)
DUX4	The DUX4 protein interacts with the cis-elements in the PITX1 promoter region, promoting PITX1 expression in response to H2O2-induced oxidative stress.	(126)
E2F1	E2F1 acts as a transcription factor by binding to the PITX1 promoter sequences 5’-GCGGCGGC-3’ and 5’-GCGGGAAG-3’, and promoting the expression of PITX1.	(2)
ERα	ERα (estrogen receptor α) and PITX1 directly interact with each other, and that PITX1 inhibits the function of ERα.	(31)
hCYP11B1	Both PITX1 and SF-1 interact and bind to and promote expression in the hCYP11B1 promoter region in the cervical cancer cell line HeLa.	(127)
IRF3 and IRF7	PITX1 binds to the promoter regions of IRF3 and IRF7, and inhibits their transcriptional activity.	(128)
LHβ	The activity of the LHβ promoter in transgenic mice is entirely dependent on the functional PITX1 regulatory element located in the proximal region of the promoter.	(129)
MTNR1A	PITX1 directly binds to the promoter region of MTNR1A, thereby promoting its expression and protecting kidney from injury.	(67)
P53	PITX1 can interact with p53 and participate in regulating cell cycle progression, apoptosis	(23)
Pan1	PITX1 collaborates with Pan1 on the insulin promoter in rats to enhance insulin secretion by transcriptional synergy.	(125)
PDCD5	PITX1 induces apoptosis by targeting the promoter of the apoptotic gene PDCD5.	(38)
POMC	PITX1 collaborates with Tpit to bind to the promoter of POMC and promote POMC expression.	(130)
RASAL1	PITX1 suppresses tumorigenesis by downregulating the oncogene RAS through RASAL1.	(24)
RHAU	The interaction between RHAU and PITX1 mRNA promotes the degradation of PITX1 mRNA.	(131)
RUNX1	RUNX1 transcription is regulated by a distal promoter that is directly inhibited by PITX1 through their interaction, thus suppressing T cell development and promoting leukemia.	(43)
SF-1	PITX1 can function as an effective ligand for SF-1 by regulating its activity in the gonadotropin pathway.	(80, 127)
Sirt1	PITX1 regulates the expression of Sirt1 by mediating the activation of the Sirt1 gene promoter.	(117)
SOX2	PITX1 interacts with SOX2 to promote squamous cell carcinoma maintenance of tumor stem cells, increasing tumor malignancy.	(36)
SOX9	PITX1 binds to the promoter region of SOX9, promoting its transcription and thus suppressing the growth and proliferation of melanoma by inhibiting SOX10 and SAMMSON.	(120)
STAT3	PITX1 directly interacts with STAT3, resulting in reduced STAT3 activity and consequent inhibition of the transcriptional activation of LINC00662.	(71)
ZCCHC10	The CCHC domain of ZCCHC10 interacts with the homologous domain of PITX1 to suppress the transcription of telomerase reverse transcriptase hTERT.	(69)

Some studies have also shown that SOX2, PITX1, and Twist1, these molecules are highly expressed in tumor stem cells of squamous cell carcinoma, while their expression levels are low in normal skin tissue (132–134). Additionally, SOX2 and PITX1 are upregulated after skin injury, which can promote cell migration and improve wound healing. This may be due to the significantly decrease in inhibitory histone H3 lysine 27 trimethylation (H3K27me3) in the promoter region in response to injury or in tumor stem cells.

Furthermore, Ana et al. demonstrated that PITX1 interacts with SOX2, TRP63, and KLF4, and PITX1 promotes the progression of skin squamous cell carcinoma (SCC) (36). Knocking down PITX1’s DNA binding domain (DBD) significantly inhibited the growth of squamous cell carcinoma, with levels of Ki67 and EdU significantly decreasing, as well as decreased SOX2 expression, but KLF4 expression levels increasing. KLF4 expression level are negatively correlated with PITX1 in SCC and suppressing the growth of squamous cell carcinoma *in vivo*. PITX1, SOX2, and TRP63 synergistically promote SCC self-renewal, cancer stem cells formation, and clonal expansion by opposing KLF4-dependent squamous cell differentiation. Overexpressing KLF4 resulted in a decrease in PITX1 and Ki67 expression levels, and an increase in FLG2 staining intensity, inhibiting tumor growth. Conversely, knocking down KLF4 resulted in an increase in Ki67, PITX1, and SOX2 expression levels, and an increase in the malignancy of tumor tissue (36).

Although most previous studies have reported PITX1 as a tumor suppressor gene that significantly inhibits the growth of cancer cells, recent research has also shown that PITX1 plays an important role in stem cells, tissue proliferation, and wound repair, suggesting that PITX1 may play a “switch” role in regulating the growth of tumor cells. Astonishingly, it has not been discussed whether PITX1 promotes angiogenesis in tumor tissue or wounds. As we mentioned above, PITX1 is necessary for cell survival, stem cell formation, and proliferation under hypoxic conditions. These interesting articles may suggest that PITX1 requires more attention in terms of angiogenesis.

## 9 Summary and outlook

In summary, PITX1 emerges as a multifaceted transcription factor, intricately interwoven into a multitude of cellular processes, exerting indispensable roles across various biological contexts. Owing to its capacity for binding to diverse gene promoters and functioning as a transcriptional regulator, PITX1 exhibits heterogeneous expression patterns across a spectrum of malignancies, frequently entailing adverse prognostic implications. Cellular and functional investigations underscore PITX1’s potential dual nature as an oncogene and a tumor suppressor, warranting comprehensive research and further exploration. On the one hand PITX1’s interactions with key genes such as P53 and RASAL1 have been shown to impede tumor proliferation, on the other hand, PITX1 has also been implicated in collaborative efforts to drive tumor progression and foster the formation of tumor stem cells in conjunction with SOX2, suggesting that the full extent of PITX1’s role remains veiled. Although PITX1 exhibits promise as a clinical tumor marker, a series of enigmas remain unsolved. Notably, (1) PITX1’s expression profiles in diverse tumors exhibit inconsistencies, with decreases observed in colon cancer and increases in lung cancer. Epigenetic modifications have been implicated in the regulation of PITX1 expression, meriting further investigation. (2) Currently, specific small molecule inhibitors targeting PITX1 have not been reported. Given PITX1’s involvement in the regulation of pivotal genes in tumor progression, such as RAS, P53, and SOX2, the development of corresponding small molecule drugs holds the potential to deepen our comprehension of PITX1. (3) Limited research has been conducted on PITX1 in mouse models of cancer, and the use of corresponding tumor models can provide better insights into the gene’s regulatory functions.

## Author contributions

All authors have substantially contributed to the work reported. Individual author contributions were as follows: conceptualization: JZ; validation: JZ and YX; writing—original draft preparation, YX; writing—review and editing, YX and JZ; supervision, YX. All authors have read and agreed to the published version of the manuscript.
